# Association Signals Unveiled by a Comprehensive Gene Set Enrichment Analysis of Dental Caries Genome-Wide Association Studies

**DOI:** 10.1371/journal.pone.0072653

**Published:** 2013-08-14

**Authors:** Quan Wang, Peilin Jia, Karen T. Cuenco, Zhen Zeng, Eleanor Feingold, Mary L. Marazita, Lily Wang, Zhongming Zhao

**Affiliations:** 1 Department of Biomedical Informatics, Vanderbilt University School of Medicine, Nashville, Tennessee, United States of America; 2 Department of Human Genetics, University of Pittsburgh, Pittsburgh, Pennsylvania, United States of America; 3 Center for Craniofacial and Dental Genetics, Department of Oral Biology, School of Dental Medicine, University of Pittsburgh, Pittsburgh, Pennsylvania, United States of America; 4 Department of Biostatistics, University of Pittsburgh, Pittsburgh, Pennsylvania, United States of America; 5 Department of Biostatistics, Vanderbilt University School of Medicine, Nashville, Tennessee, United States of America; 6 Center for Quantitative Sciences, Vanderbilt University Medical Center, Nashville, Tennessee, United States of America; 7 Department of Psychiatry, Vanderbilt University School of Medicine, Nashville, Tennessee, United States of America; 8 Department of Cancer Biology, Vanderbilt University School of Medicine, Nashville, Tennessee, United States of America; University of Southern California, United States of America

## Abstract

Gene set-based analysis of genome-wide association study (GWAS) data has recently emerged as a useful approach to examine the joint effects of multiple risk loci in complex human diseases or phenotypes. Dental caries is a common, chronic, and complex disease leading to a decrease in quality of life worldwide. In this study, we applied the approaches of gene set enrichment analysis to a major dental caries GWAS dataset, which consists of 537 cases and 605 controls. Using four complementary gene set analysis methods, we analyzed 1331 Gene Ontology (GO) terms collected from the Molecular Signatures Database (MSigDB). Setting false discovery rate (FDR) threshold as 0.05, we identified 13 significantly associated GO terms. Additionally, 17 terms were further included as marginally associated because they were top ranked by each method, although their FDR is higher than 0.05. In total, we identified 30 promising GO terms, including ‘Sphingoid metabolic process,’ ‘Ubiquitin protein ligase activity,’ ‘Regulation of cytokine secretion,’ and ‘Ceramide metabolic process.’ These GO terms encompass broad functions that potentially interact and contribute to the oral immune response related to caries development, which have not been reported in the standard single marker based analysis. Collectively, our gene set enrichment analysis provided complementary insights into the molecular mechanisms and polygenic interactions in dental caries, revealing promising association signals that could not be detected through single marker analysis of GWAS data.

## Introduction

Dental caries (also known as tooth decay or a cavity) is simply defined as a procedure that causes destruction and demineralization of hard tooth tissues such as enamel, dentin, and cementum. It is a highly pervasive chronic disease whose etiology is complex and multifactorial, with contributions from numerous factors, including microbial flora, salivary flow and composition, and fluoride exposure, among others. There has been increasing evidence of genetic components contributing to caries susceptibility [1], [2], [3], [4], [5]. Benefiting from high-throughput genotyping technologies (up to a few million single nucleotide polymorphism (SNP) biomarkers on a single chip), genome-wide association studies (GWAS) have recently been employed to search for genetic susceptibility related to dental caries [6], [7], [8], among hundreds of other complex diseases and phenotypes [9]. These dental caries GWA studies identified several loci and genes, such as *ACTN2, LYZL2,* and *AJAP1* [7], [8]. In these GWA studies, the statistical analyses of association signals are typically conducted for single markers, limiting the power to identify potential truly associated genes that may have been missed due to the multiple test adjustment necessary to control the false discovery rate (FDR). Recently, interrogating the joint effects of multiple risk loci or genes through the gene set-based analysis of GWAS data has become one popular complementary approach to single marker association tests [10]. Gene set analysis of GWAS data has been successfully applied to many diseases or traits (see recent reviews [11], [12]), including schizophrenia [13], major depressive disorder [14], type II diabetes [15], [16], [17], Crohn’s disease [18], and several types of cancer [19], [20], [21], [22]. However, to our knowledge, no such studies have been reported for gene set analysis of association data for human caries to date. In this work, we performed a comprehensive gene set analysis of GWAS data for dental caries, aiming to broaden our understanding of the role of interactions between genes for this worldwide disease.

Over the past several years, many gene set analysis methods have been proposed, which were extensively summarized in a recent review [12]. These methods address two different null hypotheses on their tests of disease associations: 1) competitive null hypothesis (Q1), which tests whether the genes within a gene set show the same association magnitude compared to the genes outside the gene set; and 2) self-contained null hypothesis (Q2), which tests whether the genes within a gene set are associated with the disease phenotype. When the real causal genes are included in only a few gene sets, the two tests may have similar results. Nevertheless, the competitive tests may be less powerful when the causal genes are shared by multiple gene sets. Apart from the difference in the null hypotheses tested, each method has its own strengths and limitations, and no single proposed strategy outperforms all the others [12].

In this study, we employed four representative methods to conduct gene set enrichment analyses for dental caries, among which two perform competitive tests (Association List Go AnnoTatOR (ALIGATOR) [23] and GenGen [24]) and the other two perform self-contained tests (SNP ratio test (SRT) [25] and the mixed model [10]). The GWAS data was collected from a recent dental caries association study [7], and the Gene Ontology (GO) annotation database [26] was the source for candidate gene sets. Our study integrated the results from different approaches and reported 13 significantly associated and 17 marginally associated GO terms. To our knowledge, this is the first comprehensive gene set analysis for dental caries, or generally for dental health, to date. Our findings provide biological insights into the potential molecular mechanisms underlying dental caries, which helps to improve our understanding of dental caries beyond the single marker level.

## Materials and Methods

### Datasets

We retrieved the dental caries GWAS data [7] from dbGaP (http://www.ncbi.nlm.nih.gov/gap) through approved access (dbGaP accession number: phs000095.v1.p1). A total of 4,020 individuals in this dataset have both genotype and phenotype data. We focused on the phenotype of ‘total primary tooth caries.’ In this dataset, the total primary tooth caries with white spots is described by the continuous variable ‘Prim_d1ft’ and the dichotomized variable ‘CAT1_PRIM_D1FT.’ By definition, individuals with disease are those with Prim_d1ft ≥ 1 (CAT1_PRIM_D1FT  =  1) and controls are those with Prim_d1ft  =  0 (CAT1_PRIM_D1FT  =  0). Subjects who were between 3 and 12 years old at the time of dental exam were included. A total of 537 cases and 605 controls, among which there are 588 males and 554 females, formed our working dataset. The samples were genotyped on the Illumina platform Human610_Quadv1_B (Illumina, Inc.). Quality checks conducted in the original study as provided by dbGaP resulted in 589,735 SNPs for the following analyses.

### Gene set annotation

The Molecular Signatures Database (MSigDB) [27] collects annotated gene sets from multiple sources. We downloaded the GO annotation [26] from MSigDB (version 3.0, C5) for gene set enrichment analysis. To avoid biological functions that are too narrowly or too broadly defined, only gene sets containing ≥ 5 and ≤ 250 genes were included in the following analyses. As a result, 1,331 GO terms passed the criteria, and the average number of genes per term was 44.

### Statistical analysis

Logistic regression was performed for association test of each of the 589,735 SNPs with CAT1_PRIM_D1FT using the GWAS analysis tool PLINK [28]. The variable “age at time of dental exam” was taken as a covariate in the regression. The overall genomic inflation factor was 1.031. We denoted the test statistic of each SNP as 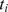 (*i* = 1,2,…,*L*, where *L* is total number of SNPs) and the p-value as 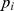 (a higher 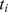 indicates a lower 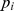). A SNP was mapped to a gene if it is located in the gene region or within 20 kb upstream or downstream of the gene. We applied this criterion based on the previous studies [23], [29], [30]. The SNP-gene mapping resulted in 20,756 protein coding genes based on the human reference assembly hg18.

Gene set enrichment studies for GWAS data have been proposed for several years. However, no single strategy outperforms all the others to date. To alleviate the potential biases in different statistical algorithms, we chose four representative methods to perform the gene set enrichment analysis in this study. These methods are GenGen [24], ALIGATOR [23], SRT [25], and the mixed model [10]. The first two methods are used to test competitive null hypothesis (Q1), while the others are used to test self-contained null hypothesis (Q2) [12]. We briefly describe the methods below. More details can be found in the original publications.

GenGen [24] is adapted from the Gene Set Enrichment Analysis (GSEA) method [27] that was originally designed to analyze gene expression data. The first step of this approach is to assign each gene a significance value 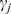 (*j* = 1,2,…,*N*, where *N* is the total number of genes) with the most significant 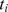 that can be mapped to this gene. Next, all the genes are ranked in descending order of 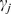, denoted by 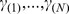. Third, for a given gene set *S* consisting of 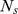 genes, an enrichment score (*ES*) is computed using a weighted Kolmogorov-Smirnov-like running-sum statistic as follows:
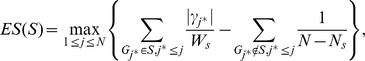



where 
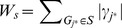
. Finally the significance of *ES(S)* is evaluated using a permutation test by shuffling the labels of cases and controls so that the linkage disequilibrium (LD) structures among SNPs are conserved.

The algorithm ALIGATOR [23] executes a SNP-based resampling procedure, which can effectively reduce the potential biases from gene size, SNP density, and LD structure. ALIGATOR defines a set of significantly associated SNPs through a predefined cutoff (e.g., p-value  =  0.05). It maps these significant SNPs to genes, which are in turn denoted as significant genes, and counts the number of significant genes for each gene set. Then, the algorithm performs a SNP-based resampling, during which SNPs are selected and mapped to genes until the number of significant genes generated by the resampling process is the same as in the original case. Resample genes are mapped to gene sets in the same way as in actual cases, and the numbers of significant genes per gene set are recorded. In our analysis, we performed resampling 10,000 times. Finally, an empirical p-value is computed for each gene set by summing the number of resampling datasets that have a higher number of significant genes than the real case.

The SNP ratio test [25] similarly defines a set of SNPs that are significantly associated with the disease through a predefined p-value threshold 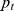. For a gene set *S*, the proportion of significant SNPs is first computed as 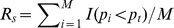, where *M* is the total number of SNPs mapped to all the genes in *S*. Then, the p-value of 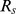 is estimated using a permutation by shuffling the case/control status among samples.

The mixed model [10] employs a hierarchical generalized linear model for gene set analysis. For each gene set, the mixed model includes the chi-square statistic (transformed from p-value) for each SNP as the outcome variable, random gene effects, and an intercept corresponding to the overall association with disease for all SNPs in the gene set. The statistical significance of the gene set is determined based on p-value for the intercept.

Both GenGen and SRT require permutation procedure by shuffling the case/control labels to determine the significance level. We generated permutation data with the same parameters and covariates 1,000 times for the use of these two algorithms. To correct multiple comparisons, the Benjamini-Hochberg method [31] was used to control the FDR.

## Results

We performed gene set enrichment analyses of dental caries GWAS data using four statistical methods (GenGen, ALIGATOR, SRT, and the mixed model) and GO annotation terms as the gene set pool. Setting FDR < 0.05 as the criterion to determine the statistical significance, the mixed model identified the largest number of GO terms that are statistically associated with dental caries, i.e., a total of 9 GO terms. The GenGen method claimed 4 significant GO terms, whereas no significant results could be found by either ALIGATOR or SRT (Tables 1 and 2). Interestingly, the GO terms identified by GenGen are all related to secretion or regulation of secretion: ‘Protein secretion,’ ‘Cytokine secretion,’ ‘Regulation of protein secretion,’ and ‘Regulation of cytokine secretion.’ The mixed model identified several GO terms that are related to neural development (‘Regulation of axonogenesis,’ ‘Regulation of neurogenesis,’ ‘Axonogenesis,’ and ‘Central nervous system development’) and three GO terms that are related to ligase activities (‘Ligase activity forming carbon nitrogen bonds,’ ‘Ubiquitin protein ligase activity,’ and ‘Small conjugating protein ligase activity’).

**Table 1:** Gene Ontology (GO) terms significantly associated with dental caries (FDR < 0.05).

GO term	# genes in term	Ratio of significant SNPs^a^	Contributing genes^b^	Method (FDR^c^)
Protein secretion	32	39/739	*ARFGAP3, LTBP2, CADM1, ABCA1, APOA1, ANG, INS, CRTAM, CARD8, CIDEA, ARFIP1, NLRP3, FOXP3, BACE2, NLRP12, GLMN, ARL4D*	GenGen (<0.001)
Cytokine secretion	18	23/468	*CARD8, CRTAM, CADM1, CIDEA, ABCA1, NLRP3, FOXP3, APOA1, INS, NLRP12, GLMN*	GenGen (<0.001)
Regulation of protein secretion	22	27/391	*CARD8, CRTAM, CADM1, CIDEA, ARFIP1, NLRP3, FOXP3, APOA1, INS, ANG, NLRP12, GLMN*	GenGen (<0.001)
Regulation of cytokine secretion	16	21/276	*CARD8, CRTAM, CADM1, CIDEA, NLRP3, FOXP3, APOA1, INS, NLRP12, GLMN*	GenGen (<0.001)
Regulation of axonogenesis	10	45/575	*RTN4, KLK8, ROBO1, MAPT, ROBO2, LRRC4C, SLIT2*	Mixed model (<0.001)
Regulation of neurogenesis	14	45/651	*RTN4, KLK8, ROBO1, MAPT, ROBO2, LRRC4C, SLIT2*	Mixed model (0.003)
Central nervous system development	122	372/6137	*GRIK1, SNCA, SHH, WNT1, PDGFC, ROBO2, UNC5C, EIF2B2, EIF2B3, SH3GL3, MDGA1, MDGA2, SH3GL2, ADORA2A, SOX3, DSCAML1, TAGLN3, SOX8, ATN1, B3GNT5, LHX6, IL1RAPL2, NKX2-2, DMBX1, JRKL, CELSR1, NEUROG3, SERPINI1, NCKAP1, S100B, MYO16, POU6F1, POU6F2, GLI2, PTEN, MBP, NDUFS4, PCP4, CNTN6, ALK, SLIT1, SLIT3, BPTF, CNTN4, SHROOM2, SHROOM4, UBE3A, ZBTB16, ALDH3A2, NPAS2, NPTX1, DNER, DCLK1, JARID2, PTPRZ1, MAL, AFF2, RCAN1, PARK2, EIF2B1, RPS6KA6, ACCN1, MAP1S, DRP2, PHGDH, PBX1, PBX3*	Mixed model (0.005)
Ligase activity forming carbon nitrogen bonds	68	111/1485	*RNF217, HLCS, MYLIP, WWP2, FBXO22, UBR3, UBE2H, BRAP, UHRF2, UBR5, UBE2M, DDB2, FBXL6, ZER1, ADSS, SYVN1, GCLC, ANAPC10, CTPS2, ASNS, PFAS, UBE2D2, FBXO3, FBXO7, CBL, MALT1, PARK2, UBE2L3, CPS1, SMURF1, PAICS, UBE2E1*	Mixed model (0.009)
Ubiquitin protein ligase activity	49	84/1175	*RNF217, MYLIP, WWP2, FBXO22, UBR3, UBE2H, BRAP, UHRF2, UBR5, UBE2M, DDB2, FBXL6, ZER1, ANAPC10, UBE2D2, FBXO3, FBXO7, CBL, MALT1, PARK2, UBE2L3, SMURF1, UBE2E1*	Mixed model (0.013)
Small conjugating protein ligase activity	51	84/1209	*RNF217, MYLIP, WWP2, FBXO22, UBR3, UBE2H, BRAP, UHRF2, UBR5, UBE2M, DDB2, FBXL6, ZER1, ANAPC10, UBE2D2, FBXO3, FBXO7, CBL, MALT1, PARK2, UBE2L3, SMURF1, UBE2E1*	Mixed model (0.014)
Glycoprotein catabolic process	12	21/267	*ADAMTS9, PSEN2, ABCG1*	Mixed model (0.031)
Axonogenesis	43	171/2782	*RTN4, NRP2, PARD3, NRP1, RTN4RL1, LRRC4C, GLI2, PAX2, SHH, ROBO1, MAPT, ROBO2, UNC5C, SPON2, PARD6B, KLK8, NRXN3, NTNG1, NTNG2, NRXN1, SLIT1, SLIT2, S100B, CYFIP1, OPHN1, CNTN4, FEZ1*	Mixed model (0.035)
Cell matrix junction	16	45/447	*PTPRC, LIMA1, BCAR1, ACTN1, ACTN2, VCL, SORBS1, LAYN, DST*	Mixed model (0.035)

a The numerator is the number of SNPs with a p-value < 0.05 from the dental caries GWAS, and the denominator is the total number of SNPs mapped to the genes in each GO term.

b Genes containing at least one SNP with a p-value < 0.05 from the dental caries GWAS are regarded as contributing genes.

c FDR adjustment is based on Benjamini-Hochberg method [31].

**Table 2:** Top 9 Gene Ontology (GO) terms identified by each of the four enrichment methods.

GenGen		ALIGATOR		SNP Ratio Test		Mixed model	
GO term	p-value (FDR)	GO term	p-value (FDR)	GO term	p-value (FDR)	GO term	p-value (FDR)
**Protein secretion (32)** a	**<0.001 (<0.001)**	Sphingoid metabolic process (12)	0.004 (1.000)	Sphingoid metabolic process (12)	0.001 (0.266)	**Regulation of axonogenesis (10)**	**<0.001 (<0.001)**
**Cytokine secretion (18)**	**<0.001 (<0.001)**	Transcription corepressor activity (93)	0.008 (1.000)	Neuropeptide receptor activity (21)	0.001 (0.266)	**Regulation of neurogenesis (14)**	**<0.001 (0.003)**
**Regulation of protein secretion (22)**	**<0.001 (<0.001)**	DNA helicase activity (24)	0.009 (1.000)	Neuropeptide binding (22)	0.001 (0.266)	**Central nervous system development (122)**	**<0.001 (0.005)**
**Regulation of cytokine secretion (16)**	**<0.001 (<0.001)**	Ligase activity forming carbon nitrogen bonds (68)	0.011 (1.000)	Translation initiation factor activity (23)	0.001 (0.266)	**Ligase activity forming carbon nitrogen bonds (68)**	**<0.001 (0.009)**
Ligase activity forming carbon nitrogen bonds (68)	0.001 (0.222)	Ubiquitin protein ligase activity (49)	0.014 (1.000)	Small protein conjugating enzyme activity (52)	0.002 (0.266)	**Ubiquitin protein ligase activity (49)**	**<0.001 (0.013)**
Positive regulation of protein secretion (12)	0.001 (0.222)	Sensory perception (188)	0.014 (1.000)	Ceramide metabolic process (11)	0.002 (0.266)	**Small conjugating protein ligase activity (51)**	**<0.001 (0.014)**
Rho guanyl nucleotide exchange factor activity (11)	0.002 (0.380)	Regulation of cytokine secretion (16)	0.015 (1.000)	Peptide receptor activity (49)	0.002 (0.266)	**Glycoprotein catabolic process (12)**	**<0.001 (0.031)**
Acid amino acid ligase activity (57)	0.003 (0.499)	ADP binding (11)	0.015 (1.000)	Translation regulator activity (38)	0.002 (0.266)	**Axonogenesis (43)**	**<0.001 (0.035)**
Monocarboxylic acid transmembrane transporter activity (11)	0.005 (0.666)	Ceramide metabolic process (11)	0.017 (1.000)	Transferase activity transferring hexosyl groups (75)	0.002 (0.266)	**Cell matrix junction (16)**	**<0.001 (0.035)**

GO terms in bold are those significant terms after adjustment for multiple testing (Benjamini-Hochberg method [31], FDR < 0.05). Underlined GO terms are those identified by more than one method.

a The number in parentheses denotes the number of genes in the corresponding GO term.

We further examined the genes that contributed to the association of these GO terms with dental caries. Genes that contained at least one SNP with its p-value < 0.05 calculated from the GWAS dataset were defined as “contributing genes.” Table 1 shows the contributing genes for the 13 associated GO terms. Some gene sets showing similar biological functions share many contributing genes. For example, ‘Regulation of axonogenesis’ and ‘Regulation of neurogenesis’ shared seven genes, including some interesting genes such as *ROBO2* and *SLIT2* (see Discussion section). Notably, the gene set ‘Cell matrix junction’ that was identified by the mixed model contains gene *ACTN2*, which was reported in the original GWAS dataset with suggestive evidence for association, but failed to meet the genome-wide significance (p-value < 10^−7^) [7]. Our finding confirmed this result based on single SNP analysis of the original GWAS data at the gene set level. To further examine whether the association of this gene set with dental caries is driven by gene *ACTN2*, we excluded this gene and performed the same gene set analysis using the mixed model approach. Interestingly, the gene set ‘Cell matrix junction’ remained significant (FDR  =  0.007) even without the gene *ACTN2*, indicating that there are additional informative genes in this gene set that contributed to the association.

Although ALIGATOR and SRT reported no significant GO terms under the criterion FDR < 0.05, several gene sets had reasonably low p-values before multiple testing correction and underwent further investigation. The high FDR values are likely due to the inherent characteristics of the algorithms used for these approaches, which is a phenomenon noticed in previous studies [29]. To better explore the results of ALIGATOR and SRT, we adopted the strategy proposed in [29]. Specifically, among the four methods we applied, the largest number of gene sets at FDR < 0.05 was 9, as reported by the mixed model approach. Therefore, we accordingly selected the top 9 gene sets ranked by their raw p-values and denoted them as candidate gene sets for each of the corresponding approaches (Table 2). Note that all the gene sets selected in this way have nominally significant p-values (within a range of 0 − 0.017). Among them, one gene set was identified by three methods, and four other gene sets were identified by two methods. Interestingly, ALIGATOR reported all five of the GO terms that can be identified by at least two strategies. Of especial note, the gene set ‘Sphingoid metabolic process’ was ranked as the most significant by the results from both ALIGATOR and SRT. The four other gene sets included ‘Ligase activity forming carbon nitrogen bonds,’ which was discovered by ALIGATOR, GenGen, and the mixed model, ‘Ubiquitin protein ligase activity’ by ALIGATOR and the mixed model, ‘Regulation of cytokine secretion’ by ALIGATOR and GenGen, and ‘Ceramide metabolic process’ by ALIGATOR and SRT. Note that GenGen and ALIGATOR are methods to investigate the competitive null hypothesis (Q1), and SRT and the mixed model are used for the self-contained null hypothesis (Q2). We saw from Table 2 that four gene sets were identified for both Q1 and Q2: ‘Sphingoid metabolic process,’ ‘Ligase activity forming carbon nitrogen bonds,’ ‘Ubiquitin protein ligase activity,’ and ‘Ceramide metabolic process.’ In total, we listed 30 top GO terms in Table 2.

In addition, we examined the set sizes (i.e., the number of genes) of the gene sets identified by each method. The sizes of the gene sets identified by the mixed model were greater than that of other methods. The median value of set sizes for the top 9 GO terms identified by the mixed model was 43, whereas the corresponding numbers were 18 for GenGen, 24 for ALIGATOR, and 23 for SRT, respectively. Meanwhile, the SNP density (represented by median number of SNPs per gene) in the GO terms discovered by four approaches are similar, i.e., 13, 12, 13, and 12 for the mixed model, GenGen, ALIGATOR, and SRT, respectively.

We further examined the association signals of the genes that resided in the 30 top GO terms reported by four different methods. A gene was considered nominally significant if it contained at least one SNP with its p-value < 0.05. Using this criterion, we found 383 nominally significant genes, among which 36 were involved in at least 4 GO terms (Table 3). The complete description of all the 383 significant genes was shown in the supplementary materials (Table S1). We used the Ingenuity Pathway Analysis (IPA, http://www.ingenuity.com, accessed in January, 2013) software to further investigate the phenotype annotations of these nominally significant genes. We searched the IPA using “dental” as the keyword in the category of ‘Functions and Diseases’ and obtained 122 related function annotation items. Eight of the 383 nominally significant genes were found in the dental related Ingenuity annotations: *PBX3, PBX1*, *BCOR*, *GLI2*, *SHH*, *DIAPH1*, *SOX3,* and *RECQL4*. They are mainly related to the Ingenuity functions ‘dental development’ and ‘dental disorder’ (Table S1).Of special note, association between *BCOR* and pit-and-fissure surface caries has been found in a recently published GWAS in the permanent dentition [6]. However, it failed to be detected in primary caries through the genome-wide, single-marker analysis approach [7].

**Table 3:** Enriched genes in the 30 top Gene Ontology (GO) terms.

Gene^a^	# terms involved	Ratio of significant SNPs^b^	Most significant SNP	p-value^c^
*PARK2*	6	28/512	rs574165	0.001
*UBR3*	6	2/36	rs16857407	0.020
*ANAPC10*	5	1/8	rs1455137	0.035
*BRAP*	5	1/11	rs10744774	0.019
*CADM1*	5	2/72	rs6589485	0.006
*CARD8*	5	5/23	rs10416565	0.008
*CBL*	5	2/13	rs2249466	0.027
*CRTAM*	5	2/22	rs3107606	0.009
*DDB2*	5	2/7	rs3781619	0.013
*FBXL6*	5	3/8	rs3817681	0.016
*FBXO22*	5	3/5	rs335675	0.011
*FBXO3*	5	1/28	rs831627	0.004
*FBXO7*	5	8/29	rs738263	0.007
*GLMN*	5	1/3	rs3103174	0.017
*INS*	5	3/9	rs11042978	0.002
*MALT1*	5	3/22	rs9783885	0.021
*MYLIP*	5	6/18	rs11969250	0.004
*NLRP3*	5	3/40	rs9988572	0.001
*RNF217*	5	2/42	rs552705	0.029
*SMURF1*	5	2/16	rs12672417	0.020
*UBE2D2*	5	2/7	rs769052	0.033
*UBE2E1*	5	4/20	rs12629302	0.008
*UBE2H*	5	1/24	rs10246707	0.024
*UBE2L3*	5	1/10	rs13054355	0.006
*UBE2M*	5	3/8	rs7249714	0.006
*UBR5*	5	4/27	rs10102559	0.003
*UHRF2*	5	1/24	rs1547258	0.030
*WWP2*	5	3/28	rs7200005	0.011
*ZER1*	5	1/4	rs10988111	0.017
*APOA1*	4	1/6	rs10047459	0.015
*CCKBR*	4	2/19	rs2880829	0.004
*CIDEA*	4	1/16	rs8084404	0.040
*FOXP3*	4	1/7	rs5906761	0.016
*NLRP12*	4	2/19	rs7259148	0.005
*ROBO2*	4	15/171	rs9836971	0.005
*SLIT2*	4	6/106	rs12503652	0.003

a Genes that have at least one SNP with a p-value < 0.05 and are involved in at least 4 gene sets were listed.

b The numerator is the number of SNPs with a p-value < 0.05 in a gene and the denominator is the total number of SNPs mapped to the gene.

c The p-value of the most significant SNP in the gene.

## Discussion

With many GWAS datasets having been released, gene set enrichment analysis was proposed as an important and complementary approach to the traditional single marker analysis of GWAS data. Compared to single marker analysis, gene set analysis focuses more on biological functions of gene products as well as their regulation in the cellular systems. Thus, this strategy has advantages in revealing potential molecular mechanisms underlying diseases or traits. In addition, both real and simulation studies indicated that gene set enrichment analysis could increase the power of detecting association signals [10], [19]. In this study, we conducted a comprehensive gene set analysis for dental caries GWAS data [7]. Applying four methods (GenGen, ALIGATOR, SRT, and the mixed model), we identified 30 GO terms that were significantly or marginally associated with dental caries (Table 2). Among them, five gene sets were identified by at least two enrichment methods (i.e. ‘Ligase activity forming carbon nitrogen bonds,’ ‘Regulation of cytokine secretion,’ ‘Ceramide metabolic process,’ ‘Sphingoid metabolic process,’ and ‘Ubiquitin protein ligase activity’). While definitive roles for the gene sets cannot be identified as sufficient to cause cariogenesis, the five GO terms are plausible factors for disease. These terms encompass broad functions that potentially interact and contribute to the oral immune response to caries-related organisms. The oral environment contains bacteria that may lead to a host inflammatory response eliciting cytokines [32], [33], [34]. This inflammatory response involves the sphingolipds, of which sphingoids and ceramides are constituent components released during the response [35], [36]. Anaerobic organisms present in the oral cavity thrive under hypoxic conditions, which have been observed to stimulate cytokine production regulated by ubiquitin protein ligases [33], [37], [38], [39]. While no direct action from carbon nitrogen bond ligases is identified within the immune response pathways, it is possible that they function in a parallel maintenance mechanism for the immune-related pathways.

We further investigated the 36 identified genes associated with the 30 top GO terms (Table 3) for their potential overlap with caries development. For each gene, the GeneCards (http://www.genecards.org/) entry (summaries and function) and OMIM (http://omim.org/) entry were queried to summarize gene functions. GeneCards aliases were also searched for in OMIM. A query for gene name and each alias cross-listed with “caries”, “tooth”, and “dentin” was conducted in PubMed to further assess known genetic roles related to dental caries.

Based on our gene-based literature search, five genes from either ligase activity (gene: *WWP2*, *RNF217*), neuronal development (gene: *ROBO2*, *SLIT2*), or cytokine/protein secretion (gene: *INS*) gene sets listed in Table 2 might be potentially associated with dental traits. Only the cytokine/protein secretion term was identified by more than 2 gene set enrichment methods. WWP2 is a member of ligase activity pathways and functions as a ligase for and mediates degradation of PTEN, whose gene is expressed in mouse oral development [40], [41]. *RNF217* is located at 6q22.31, a genomic region reported to be associated with oral cleft [42]. ROBO2 is a receptor for SLIT2 and possibly SLIT1. SLIT1 and SLIT2 appear to work cooperatively to establish anatomical midlines during neuronal development and establishment of olfactory organization [43]. Gene *SLIT1* is also expressed in the primary and secondary enamel knots during molar tooth cusp formation [44]. INS may impact caries through insulin sensitivity [45] or more controversially through the activation of dentin-related genes [46], [47]. Insulin receptor binding sites are present on rat incisors [48]. None of these relationships are “smoking guns” for caries development, but the gene sets and the subset of tooth-related genes raise interesting possible mechanisms for caries. These contributing genes encompass multiple functions or biological processes related to tooth development or dental caries, suggesting that our gene set enrichment analysis was effective and the findings were insightful to the understanding of molecular mechanisms of disease at the system level.

Although the genetic research has been applied to dental caries for a long time (see a recent review [49]), interpretation of the results remains challenging. In our gene set enrichment analysis, few GO terms or genes we identified exhibit explicit roles for caries development. One possible reason is the complex characteristics of dental caries. While many caries risk factors have been reported, few of them have been rigorously replicated or confirmed [8]. Thus, the predefined gene sets may be too general to play definitive functions in cariogenesis.

In this study, four popular gene set analysis methods, i.e., GenGen, ALIGATOR, SRT, and the mixed model, were applied to a real GWAS dataset. Although our primary interest is to unveil the genetic components of dental caries, these results also provided a comprehensive benchmark resource to compare these methods. We only observed limited consistency among the outputs of different algorithms. The inconsistency is not unexpected, mainly because different methods employ different intrinsic strategies and may test different null hypotheses (i.e., competitive vs. self-contained null hypothesis). In addition, different ways to preprocess GWAS data might influence the enrichment results. For example, one important step in performing gene set analysis of GWAS data is to map SNPs to genes and compute a gene-based statistical value. Typically, only a subset of SNPs within a gene plays roles in the disease, yet taking all the SNPs into account will likely reduce the test power. However, in practical applications, it is difficult to find the most relevant SNPs for gene set analysis. Many approaches, like GenGen, denote the most significant SNP as gene’s representative, which may exclude important additional SNPs if a gene has more than one association signal. Using ALIGATOR, all SNPs mapped to a gene are consulted, and a gene is defined as significant if it harbors at least one nominally significant SNP, requiring a predefined threshold that may be chosen arbitrarily. Therefore, the analysis results from ALIGATOR could be sensitive to the choice of threshold in different data sets [23]. Similarly, in the SRT method, all SNPs mapped to a gene are considered, and this approach also requires a preselected threshold to define the associated SNPs. One advantage in SRT is its incorporated permutation test by randomly swapping case/control labels among samples to reduce the sensitivity driven by the choice of threshold. In contrast, the mixed model approach accounts for the p-values of all the SNPs mapped to a gene without requiring predefined thresholds. Thus, this method avoids potential arbitrary definitions and quantitatively leverages the information of all SNPs.

One limitation in this study is the FDR values attained using the four methods are quite different from each other. The top 9 GO terms identified by ALIGATOR had an FDR value of 1. The situation is better in SRT, but the top GO terms also hardly reach a noteworthy FDR significance level. The high FDR could be the result of several factors. One is the inherent drawbacks of the tools used. For example, two GO terms, ‘Ligase activity forming carbon nitrogen bonds’ and ‘Ubiquitin protein ligase activity,’ were ranked as the fourth and the fifth most significant gene sets, respectively, in both results by ALIGATOR and the mixed model. However, their FDR values differed substantially in the two results. Another possible reason for this high FDR might be attributed to the incomplete information in the current annotation databases, especially for some phenotypes without much molecular biology knowledge. In contrast to most common diseases such as cancer, the functional annotation for dental caries has been very limited so far. In fact, we also performed a gene set enrichment analysis using the canonical pathways from KEGG [50], a widely used pathway database. There were only a small number of KEGG pathways eligible for our analysis (181 pathways with ≥ 5 and ≤ 250 genes), and none were significant KEGG pathways at FDR < 0.05. The failed detection of promising pathways for dental caries reflected that most, if not all, genes in the current version of the KEGG database are not thoroughly annotated. Another limitation in pathway annotations is that we used an old version of the GO term set (MSigDB, version 3.0, C5). New versions of GO data were released during our data analysis, which now included more than 10,000 GO terms (06/26/2013 release). However, major efforts are needed to process the redundancy of genes in GO terms, as processed in version 3.0, C5, to avoid an over-adjustment through multiple testing correction. This work, as well as more robust pathway enrichment analysis in future, may help better define dental caries pathways.

In summary, we applied four representative gene set enrichment analysis methods to currently available dental caries GWAS data. Our work, to date, is the first gene set enrichment study for this worldwide disease. We reported 13 significantly associated and 17 marginally associated GO terms as likely involved in dental caries via their gene functions. The findings provided insights and interpretations into the underlying biological process for dental caries. Our study mainly focused on genetic signals in GWAS data. In future work, an integration of other genetic and genomic information (such as gene expression, linkage scan and protein-protein interaction network [51], [52], evidence from multiple species [53], and multi-dimensional functional module analysis [54]) may open new avenues to understand the etiology of dental caries.

## Supporting Information

Table S1 **List of genes that are nominally significant with dental caries in the 30 top GO terms.** This table includes 383 nominally significant genes that appeared in the top 30 GO terms shown in Table 2. Genes that have at least one SNP with a p-value < 0.05 are regarded as nominally significant genes (without multiple testing correction).
(XLSX)
